# The Enigma of Atypical Cartilaginous Tumors: Surgery or Surveillance?

**DOI:** 10.3390/cancers15194696

**Published:** 2023-09-23

**Authors:** Andreas Leithner, Maria Anna Smolle

**Affiliations:** Department of Orthopaedics and Trauma, Medical University of Graz, Auenbruggerplatz 5, 8036 Graz, Austria; maria.smolle@medunigraz.at

During the last 20 years, the treatment of atypical cartilaginous tumors (ACTs) of the long bones has undergone a dramatic change: while these formerly called chondrosarcomas G1 previously led to wide resections and big reconstructions with megaprostheses, today, the use curettage of the lesions and filling the defect e.g., with bone cement, is widely accepted. This procedure leads to an acceptable local recurrence rate between 0% and 11% [1,2,3] besides a nearly non-existent metastatic potential [2,4,5].

The current discussion goes even further: strengthened by publications such as the current manuscript by Decker C et al. (Cancers 2023) [6], several centers started to use active surveillance with MRI for both enchondromas (EC) and ACTs. However, there is a substantial risk of progression (13% in the current series (Decker C et al. Cancers 2023)) and a reported risk of malignant transformation of ACTs to high-grade chondrosarcoma of 1–6% [7,8].

Therefore, international treatment guidelines are needed. For those, several questions have to be answered:

1. Can we differentiate EC from ACT based on radiology only? Until recently, the diagnosis of an ACT was primarily based on histology—the detection of bone entrapment as a clear sign of infiltrative growth was the main factor leading to a malignant diagnosis. However, as we tend to be more reluctant concerning operations, which radiological signs help us to define which lesions we should follow up more closely and which lesions we should definitely operate on? What about perifocal edema, deep endosteal scalloping, lack of calcification over time, and growth within a certain time period?
2. Can we just observe suspected ACTs? If yes, in which intervals? Besides the well-known indicators of high-grade malignancy such as extra-osseous components, are there clear red flags indicating that we should perform surgery? If the lesion is evidently growing (e.g., over 10% in maximal diameter in 6 months), is that a clear indication for surgery? Furthermore, should, as the authors of the present manuscript mention, pain and age be regarded as two other relevant factors?
3. Is the incidence of ACTs really rising? Although some reports claim a dramatic increase in the incidence of ACTs [8], larger studies reported a quite stable situation with a prevalence of 1.45% for benign and intermediate cartilaginous tumours at the knee joint and 0.43% at the shoulder joint, nearly exclusively detected as incidental findings [9,10]. The suspected increase might reflect an increased awareness and/or a faster radiological classification of chondroid lesions as ACTs.

To summarize, we need an international consensus on the diagnosis and treatment of ECs and ACTs, ideally supported by a predictive nomogram. To create (and to test) this, we need more large, observational, ideally multicentric, long-term follow-up studies. Additionally, as the diagnosis of dedifferentiated sarcoma is dismal, we should search for early signs of dedifferentiation in order to treat these patients in as timely a manner as possible.
